# Secondary tuberculosis of adjacent segments after anterior cervical discectomy and fusion: A case report

**DOI:** 10.3389/fsurg.2022.1077353

**Published:** 2023-01-06

**Authors:** Chengjiang Liu, Yidong Liu, Boyuan Ma, Mengmeng Zhou, Xinyan Zhao, Xuanhao Fu, Shunli Kan, Wei Hu, Rusen Zhu

**Affiliations:** Department of Spine Surgery, Tianjin Union Medical Center, Tianjin, China

**Keywords:** tuberculosis, anterior cervical discectomy and fusion, adjacent segment infection, cervical spine, cervical spondylotic myelopathy

## Abstract

**Introduction:**

Anterior cervical discectomy and fusion (ACDF) is a common operation for spinal surgery to treat a variety of cervical diseases. The postoperative infection rate of this procedure is extremely low, and adjacent segments are rarely involved. Tuberculosis (TB) is a common infectious disease that affects the spine in less than 1% of cases and is more common in the thoracolumbar and rarely cervical spine. Herein, for the first time, we report tuberculosis infection in adjacent segments after ACDF.

**Case presentation:**

We report a 50-year-old patient with cervical spondylotic myelopathy (CSM) who was discharged from the hospital after receiving ACDF at the C3/4 level. Two months later, he was admitted to the hospital with neck pain and found to be infected with tuberculosis in C4/5. After 4 months of anti-tuberculosis treatment, the vertebral body was fused.

**Conclusion:**

After ACDF, the adjacent cervical vertebrae were infected with TB but the infection was limited. We believe that the special vertebral blood supply and postoperative secondary blood-borne infection may lead to the occurrence of extrapulmonary tuberculosis.

## Introduction

Tuberculosis (TB) remains the main cause of death around the world, with a prevalence of 900,000 cases and mortality of 150,000 cases each year (1). The most common extra-pulmonary tuberculosis musculoskeletal site is the spine (approximately 1%–2% of all cases) (2), with the predilection for the thoracic and lumbar regions (3). Cervical Spine Tuberculosis (CST) is relatively rare but usually leads to cervical instability and severe neurological deficits. Cervical tuberculosis can lead to severe neurological damage, cervical instability and nerve root and vertebral artery involvement, and these complications are extremely disabling and lethal (4). The early diagnosis and management of CST are very important for preventing those debilitating complications (5). Herein, we report a case of a middle-aged man who had CST after anterior cervical discectomy and fusion (ACDF). ACDF is widely used for cervical spine degenerative diseases. This procedure is well-developed and the postoperative infection rate is very low. The infected segments are overwhelmingly surgically invasive segments and the most common microorganisms associated with infection are Staphylococcus aureus and Staphylococcus epidermidis (6–9). To our knowledge, this is the first report of adjacent-segment tuberculosis infection after cervical spine surgery.

## Case presentation

**Figure 1:**
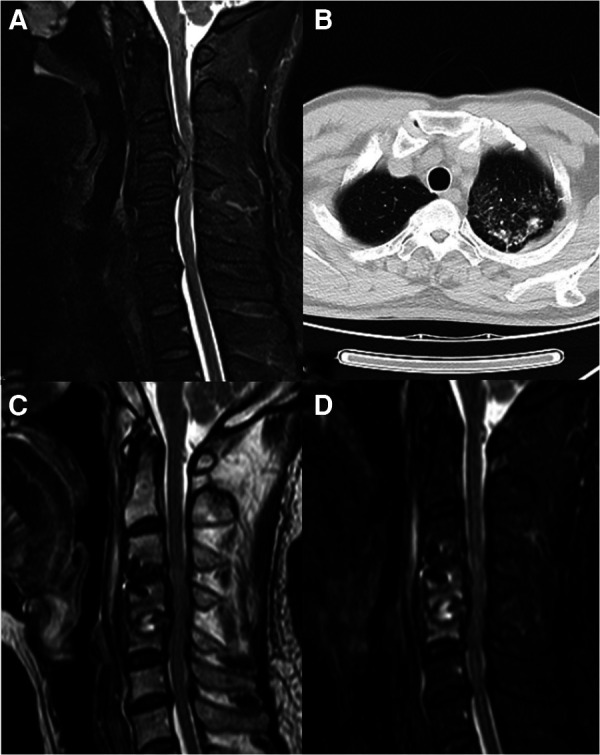
(**A**) Preoperative T2. (**B**) Preoperative chest CT. (**C**) Two months postoperative T2. (**D**) Two months postoperative fat reduction image.

**Figure 2:**
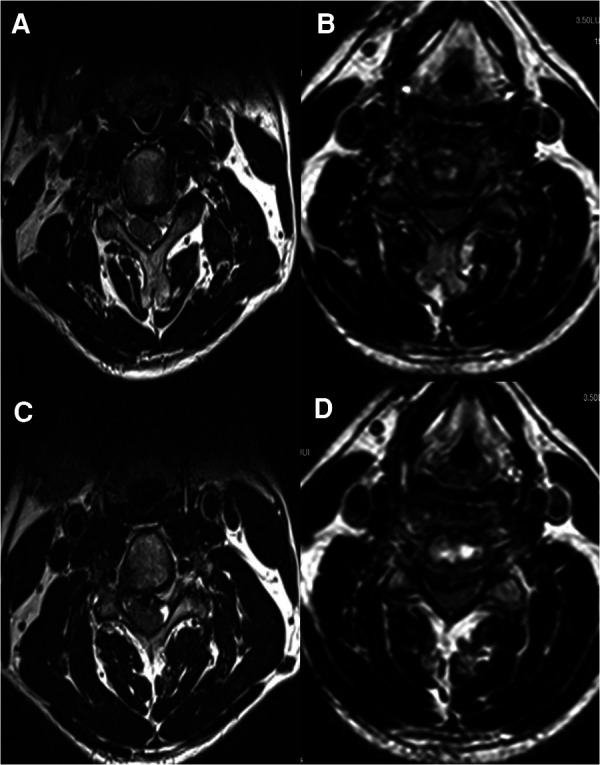
(**A**) Preoperative C3/4 MRI cross-section. (**B**) Postoperative C3/4 MRI cross-section. (**C**) Preoperative C4/5 MRI cross-section. (**D**) Postoperative C4/5 MRI cross-section.

**Figure 3:**
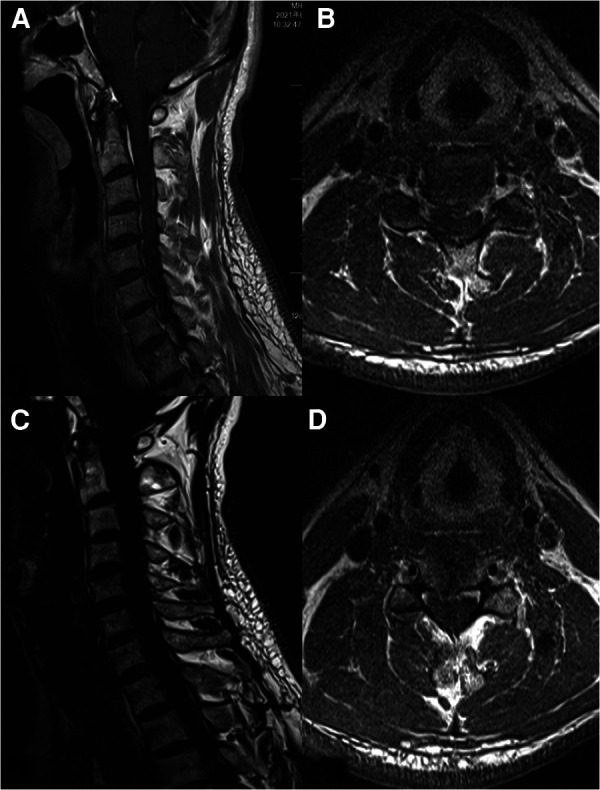
(**A**) Preoperative sagittal enhancement MRI of the cervical spine. (**B**) Preoperative C3/4 cross-sectional enhanced MRI. (**C**) Fat suppression of preoperative sagittal enhancement MRI. (**D**) Preoperative C4/5 cross-sectional enhanced MRI.

**Figure 4:**
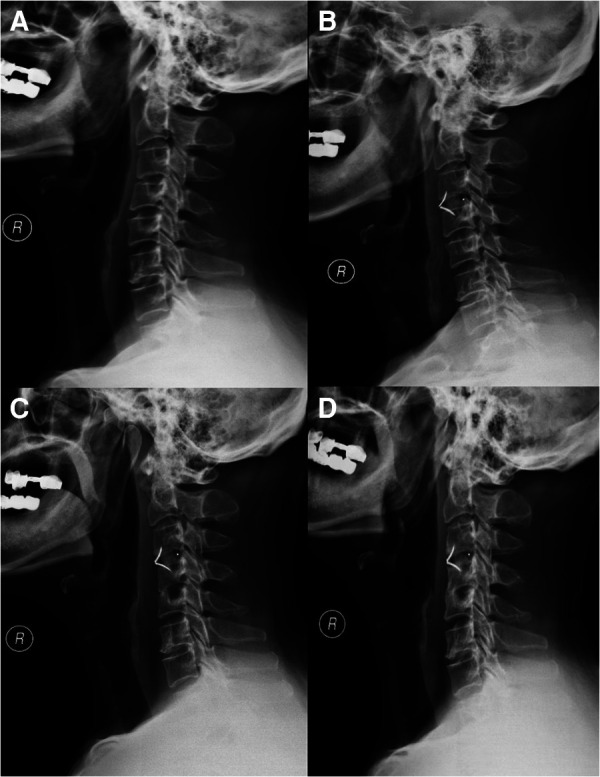
(**A**) X-ray before operation. (**B**) X-ray 2 days after the operation. (**C**) X-ray 2 months after the operation. (**D**) X-ray 12 months after the operation.

A 50-years-old retired man came to our institution with a 1-year history of neck stiffness and muscle weakness. He reported the feeling on the cotton while walking and the patient's bilateral tendon hyperreflexia and Hoffmann's sign were positive. His family and medical history were not significant. Laboratory results showed a normal erythrocyte sedimentation rate (5 mm) and C-reactive protein (1.2 mg/L). The cervical spine Magnetic resonance imaging (MRI) demonstrated that the cervical disc at the level of C3/4 was herniated to the left from the median, and the spinal cord was obviously compressed (Figures 1A, 2A,C). Chest CT showed an old foci of pulmonary TB in the upper lobe of the left lung (Figure 1B). A preoperative enhanced MRI ruled out the possibility of a tumour in the patient's cervical spine (Figures 3A–D). Thus, the patients were considered to be cervical spondylotic myelopathy (Figure 4A).

**Figure 5:**
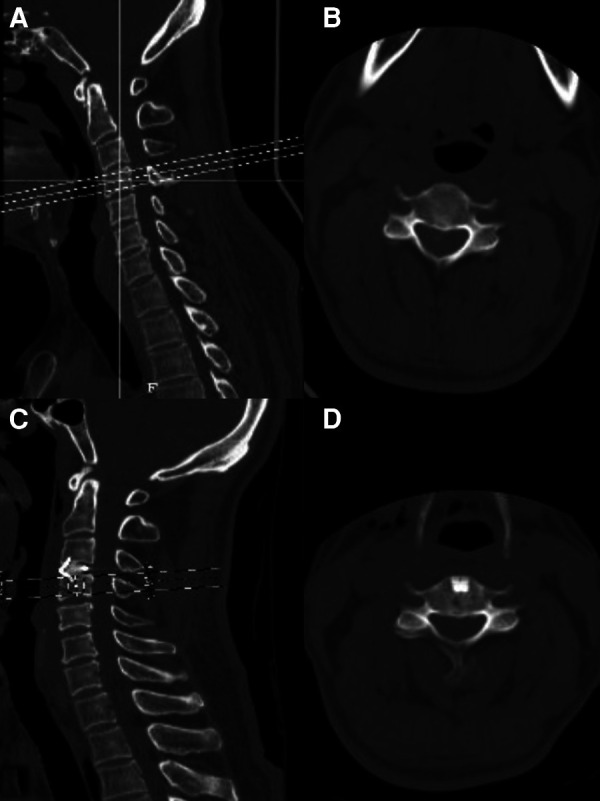
(**A**) Preoperative C3/4 CT sagittal plane. (**B**) Preoperative C3/4 CT cross-section. (**C**) Postoperative C3/4 CT sagittal plane. (**D**) Postoperative C3/4 CT cross-section.

The patient then underwent an anterior cervical discectomy and fusion (ACDF) at the C3/4 segment in hope of a cure. The patient's herniated cervical disc was carefully removed and the bleeding was stopped. The implant was allogeneic bone-filled polyetheretherketone (PEEK). On the second day postoperatively, an x-ray examination (Figure 4B) confirmed the decompression of the spinal cord, and the implant structure was stable and the position was correct. Postoperative MR of the patient's C3/4 and C4/5 segments showed good postoperative decompression and no vertebral body infection (Figures 2B,D). Postoperative laboratory results showed normal erythrocyte sedimentation rate (5 mm) and C-reactive protein (4.1 mg/L). The preoperative CT showed no infection in the cervical vertebrae (Figures 5A,B). The postoperative cervical spine CT showed successful surgical decompression, stable endophytes and no signs of infection in the vertebral body (Figures 5C,D). One week postoperatively, the patients didn't show any complaints and can walk independently when leaving the hospital.

However, 2 weeks postoperatively, the patient developed neck pain, which was worsened when lying down and turning to the left. He came to our department again on 2021.8.16. Since the onset of the disease, The patients did not show any symptoms of TB infection such as low-grade fever, night sweats, occasional fatigue, insomnia, and no obvious abnormality in limb movement and sensation. The cervical spine MRI imaging 2 months after surgery (Figures 1C,D, 4C) showed bone erosion at the C4/5 vertebral body edge with swelling of the paravertebral soft tissue, and inflammatory manifestations of the C4/5 intervertebral disc and vertebral body. The patient's pain was not relieved after 48 h of empiric antibiotic (ceftriaxone) treatment. Although vancomycin was used to upgrade the antibiotics, after 48 h, there was still no obvious improvement. The elective surgery was arranged for the patient immediately. Six hours before the operation, the patient reported insomnia and night sweats for the past 2 days. After follow-up, the patient was found to have a low fever. New assay results showed neutrophil percentage 52.10%; lymphocyte percentage 41.1%; tuberculosis -infected T cell culture (TBA: 13sfc/2.5*10e5; TBB: 6 sfc/2.5*10e5) was positive and IFN release was detected -γ-specific lymphocytes.

After out-of-hospital consultation, the patient was diagnosed with cervical spine TB, and received anti-tuberculosis drug treatment: isoniazid (INH) 0.3 g QD, rifampicin (RMP) 0.45 g QD, ethambutol (EMB) 0.75 g QD, levofloxacin 0.5 g QD. One-week post-treatment, the patient's neck pain was relieved, and he requested to be discharged home for treatment after informed consent. The patient was instructed to continue the course of anti-tuberculosis treatment, with cervical collar fixation to avoid activities involving neck movement and weight-bearing for 3 months.

The patient was kept regular follow-ups. On June 16, 2022, the patient underwent a cervical spine x-ray examination and the results showed (Figure 4D) that the C3/4 and C4/5 segments were fused, and the paravertebral soft tissue swelling disappeared. The patient's neck pain improved significantly, and it is recommended to continue anti-tuberculosis treatment.

## Discussion

The ACDF surgical sites are highly vascular. However, the postoperative vertebral infection has been very rare in the last decades because of the widespread use of various antibiotics. Through the literature retrieval, the overall infection rates were around 0.1%–1.6% (10), with a lower incidence of concomitant adjacent segment infection, but an increased incidence of neurological damage following the formation of an intraspinal or paravertebral abscess (11). Bone TB involving the spine is more common in the thoracic and lumbar spine, and cervical spine TB accounts for about one-fifth of spinal TB cases (3). Compared with thoracic and lumbar TB, cervical TB infection can lead to extensive destruction of bone and ligaments in the infected sites, resulting in the spine column instability and compression of the spinal cord. Therefore, early and accurate diagnosis has important clinical significance for the prognosis and development of patients. The imaging examination of this patient showed inflammatory manifestations of the C4/5 intervertebral disc and vertebral body, and postoperative infection was considered (12). We have found that diagnostic treatment with anti-tuberculosis drugs is clearly effective and can be continued in accordance with treatment principles without the need for further surgery (13).

The peculiarity of this case is that the segment infected with TB after surgery is adjacent. Analyzing the causes of TB infection in adjacent segments, we proposed the following hypotheses:

1. The particularity of the blood supply of the cervical spine.

Tuberculosis of the spine often develops when Mycobacterium tuberculosis reaches the vascular plexus of the cancellous bone of the vertebral body *via* a hematogenous route of dissemination. The main site of primary infection is usually the pulmonary region and is transmitted by arterial or venous routes (14). The subchondral region of each vertebral body is rich in the vascular plexus of Batson's veins (15). The venous plexus of Batson is a valveless system that allows blood to flow freely in both directions. The venous plexus of Batson can be a potential dissemination route for the development of spinal TB (16). Anterior nutrient vessels in the front of the cervical spine all pass through the long neck muscles, forming vascular bundles in the prevertebral fascia and penetrating the vertebra from the anterior nutrient foramen (17). The stretch on the longus cervicalis during surgical exposure obstructs blood flow to the anterior vertebral body. Extensive removal of the vertebrae in the vicinity of the superior endplate during further procedures may lead to direct damage to the feeding vessels that nourish the superior endplate. Injury to the blood vessels may result in slow blood flow to the vertebral body or even impeded return flow, leading to the accumulation of pathogenic bacteria. In addition, the fusion of C3 and C4 must be established on the resection of the C3/4 intervertebral disc. This means that the blood supply of C3 and C4 after fusion is more abundant than that before surgery because the intervertebral disc in adults is bloodless. The richer blood supply of the C4 vertebral body than that prior to the surgery may also be a potential reason for promoting the spread of Mycobacterium TB to the vertebral body.

2. Surgery-induced immunosuppression.

Characteristics of people commonly susceptible to TB include chronic nutritional deficiencies, poverty, peripheral vascular disease, immunosuppressive therapy and immunodeficiency diseases (18). Although ACDF surgery is less traumatic, it can also temporarily reduce the body's immunity. Because surgery and anesthesia can lead to various metabolic reactions, resulting in a systemic immunosuppressive state after surgery, which may lead to the recurrence of extrapulmonary TB in patients (19). At the same time, to reduce the stimulation of the spinal cord caused by the discectomy, 80 mg of methylprednisolone sodium succinate was intravenously administered to the patient during the operation. The patient had ruled out other predisposing factors for infection, such as diabetes, malnutrition, HIV infection, chronic steroid use, or alcohol abuse. Although the activity status of Mycobacterium TB has not been detected, this may be the most likely route.

3. Implant contamination directly infects adjacent segments during surgery.

Accidental inoculation of bacteria into the C4 vertebral body following contamination of the needle used to locate the surgical segment under x-ray before surgery. When we reviewed the surgical records, it was clear that the needle was placed in the C3–4 disc, so this possibility was ruled out. In addition, contamination of the Casper needle used in the procedure may also be a potential cause (20).

## Conclusion

The incidence of TB infection in patients after ACDF is extremely low. The possibility of infection should be considered once the symptoms change after the initial surgery. When empirical antibiotic therapy is not effective, apart from the laboratory and imaging results, more attention should be paid to the patient's changing symptoms. Early and accurate diagnosis of TB is critical to the efficacy of treatment. TB infection of adjacent segments after cervical spine surgery is extremely rare, which may be due to the particularity of the blood supply of the vertebral body and the changes in the blood supply of the vertebral body after surgery, resulting in the hematogenous dissemination of Mycobacterium TB.

## Data Availability

The original contributions presented in the study are included in the article/Supplementary Material, further inquiries can be directed to the corresponding author.
